# Uncertainty-aware automated labeling of intracranial arteries using deep learning

**DOI:** 10.1186/s12880-026-02276-5

**Published:** 2026-03-16

**Authors:** Javier Bisbal, Patrick Winter, Sebastián Jofre, Aaron Ponce, Sameer A. Ansari, Ramez Abdalla, Michael Markl, Oliver Welin Odeback, Sergio Uribe, Cristian Tejos, Julio Sotelo, Susanne Schnell, David Marlevi

**Affiliations:** 1 Department of Molecular Medicine and Surgery, Karolinska Institutet, Stockholm, Sweden; 2 Biomedical Imaging Center, Pontificia Universidad Católica de Chile, Santiago, Chile; 3 Department of Electrical Engineering, School of Engineering, Pontificia Universidad Católica de Chile, Santiago, Chile; 4 Millennium Institute for Intelligent Healthcare Engineering (iHEALTH), Santiago, Chile; 5 Departamento de Informática, Universidad Técnica Federico Santa María, Santiago, Chile; 6 Escuela de Ingeniería Civil Informática, Universidad de Valparaíso, Valparaíso, Chile; 7 Department of Medical Imaging and Radiation Sciences, Faculty of Medicine, Nursing and Health Sciences, Monash University, Melbourne, Australia; 8 Department of Medical Physics, Institute of Physics, University of Greifswald, Greifswald, Germany; 9 Institute for Medical Engineering and Science, Massachusetts Institute of Technology, Cambridge, MA USA; 10 Department of Radiology, Northwestern University, Chicago, IL USA; 11 Department of Neurological Surgery, Northwestern University, Chicago, IL USA; 12 Department of Neurology, Northwestern University, Chicago, IL USA

**Keywords:** Intracranial artery labeling, 3D ToF-MRA, UNet, Intracranial 4D flow MRI, Uncertainty quantification, Test time augmentation

## Abstract

**Background:**

Accurate anatomical labeling of intracranial arteries is critical for cerebrovascular diagnosis and hemodynamic analysis, but remains time-consuming and prone to inter-operator variability. While deep learning provides an automated solution, its clinical adoption is limited by the lack of confidence measures. Incorporating uncertainty quantification into automated labeling could enhance interpretability by identifying ambiguous or abnormal regions and support clinical trust, yet this aspect remains underexplored.

**Methods:**

To address this gap, we introduce an uncertainty-aware deep learning framework for automated artery labeling from 3D Time-of-Flight Magnetic Resonance Angiography (3D ToF-MRA) segmentations (*n* = 35). Three convolutional neural network architectures were evaluated: (1) UNet with residual encoder blocks, (2) CS-Net, an attention-augmented UNet with spatial attention, and (3) nnUNet, a self-configuring framework that adapts architecture and training to dataset characteristics. Confidence was modeled via test-time augmentation (TTA) combined with a novel coordinate-guided strategy to reduce interpolation errors during inference. Generalizability was assessed by evaluating a subset of the public TubeTK ToF-MRA dataset (*n* = 20).

**Results:**

Voxelwise uncertainty maps highlighted anatomical ambiguities, pathological variations, and inconsistencies in manual references, providing intuitive confidence indicators. nnUNet achieved the highest performance (average Dice score 0.93; clDice 0.94; average surface distance 0.35 mm; 95th percentile of Hausdorff distance 4.51 mm), demonstrating robustness in complex vascular regions. On the TubeTK dataset, nnUNet maintained robust generalization (average Dice score 0.87; clDice 0.87; average surface distance 0.42 mm; 95th percentile of Hausdorff distance 5.85 mm). Validation against co-registered 4D flow MRI showed close agreement between flow velocities derived from automated and manual labels, with no significant differences.

**Conclusion:**

The proposed framework delivers a scalable, accurate, and uncertainty-aware solution for intracranial artery labeling. By integrating uncertainty quantification, it offers a transparent and clinically trustworthy tool to facilitate cerebrovascular imaging workflows and support subsequent hemodynamic analyses.

## Introduction

The intracranial arterial system plays a critical role in brain perfusion to maintain normal cognitive function. Occlusion or stenosis of these blood vessels can cause vascular alterations that contribute to the development of cerebrovascular or neurodegenerative diseases  [1, 2].

Three-dimensional Time-of-Flight Magnetic Resonance Angiography (3D ToF-MRA) is the clinical gold standard for non-invasive imaging of the intracranial vasculature. Recently, 4D flow MRI has emerged as a new modality, adding valuable functional hemodynamic data, including regional blood flow variations. In particular, intracranial 4D flow MRI has shown promise in assessing a variety of vascular pathologies, including aneurysms  [3–5], arteriovenous malformations  [6, 7], and intracranial atherosclerotic disease (ICAD)  [8, 9].

Accurate quantification of 4D flow MRI data is highly dependent on both segmentation and precise anatomical labeling of the intracranial arteries  [10, 11]. Although several methods have been proposed for automated segmentation of the vascular tree  [12–15], our study focused on automated labeling of the major vascular structures. This process remains one of the most labor intensive and clinically critical tasks in cerebrovascular imaging and is often significantly affected by interoperator variability  [11].

To achieve automated vessel labeling, traditional graph-based approaches model the vascular tree using relational graphs derived from the centerlines  [16–18]. However, their performance is often compromised in cases of severe stenosis or disconnected vasculature. Moreover, these methods typically overlook contextual information embedded in the 3D image space.

Deep learning (DL) models offer an alternative by learning vessel features directly from image data. These models leverage structural and spatial context to produce anatomically consistent labeling results. Most existing works rely on UNet-derived architectures  [19–23], which offer robust baselines but lack systematic comparisons between architectural variations. Furthermore, they do not provide any quantification of model confidence or predictive reliability. This limitation poses a key barrier to clinical translation, as uncertainty awareness is essential for the interpretability, trust, and safe deployment of deep learning models in medical imaging.

Recent studies have incorporated uncertainty estimation into cerebrovascular segmentation frameworks  [24, 25]. For instance, Rathore et al.  [24] introduced an ensemble-based approach for estimating uncertainty in brain-vessel segmentation, showing that regions of high uncertainty often coincide with segmentation errors and out-of-distribution data. Osman et al.  [25] proposed a semi-supervised strategy with dual uncertainty estimation to enhance vessel delineation in 3D ToF-MRA. However, intensity-based features and their derived uncertainty are susceptible to domain shifts across scanners and acquisition protocols, which can degrade both segmentation performance and the trustworthiness of uncertainty estimates  [26–28]. Moreover, uncertainty derived directly from ToF-MRA images can be confounded by surrounding non-vascular tissues, which may introduce irrelevant contextual information.

To address these gaps, we developed an uncertainty-aware deep learning framework for automated labeling of intracranial arteries from 3D ToF-MRA segmentations. Our contributions are threefold. First, we evaluate and adapt two robust deep learning architectures, nnUNet and CS-Net, for intracranial artery labeling, and demonstrate improved accuracy compared to a UNet-based architecture. nnUNet achieves this through a self-configuring framework, which derives a dataset “fingerprint” (e.g., voxel spacing, image dimensions, and label characteristics), and uses a rule-based configuration to automatically select key pre-processing and training hyperparameters (e.g., image resampling strategy, network topology)   [29]. On the other hand, CS-Net introduces spatial attention mechanisms designed for curvilinear structures  [13]. Second, we introduce uncertainty estimation for intracranial artery labeling via test-time augmentation (TTA)  [30] directly from labeled segmentations, rather than from intensity-based features, thereby reducing sensitivity to scanner- and protocol-dependent intensity variations and limiting the influence of unrelated anatomical regions  [31]. Third, we introduce a coordinate-guided strategy to invert TTA transformations, reducing interpolation errors when mapping augmented predictions back to the original space and mitigating boundary artifacts that can distort uncertainty estimates.

Together, these contributions yield a robust and interpretable framework for intracranial artery labeling that improves labeling accuracy while providing uncertainty estimates that highlight ambiguous or inconsistent regions, supporting more trustworthy cerebrovascular analysis.

## Methods

### Study cohort

We retrospectively selected 25 patients (11 females) from an IRB-approved ICAD study at Northwestern Memorial Hospital. Fourteen cases exhibited severe stenosis, defined as constriction > 70%, while the remaining cases showed moderate stenosis, with constriction ranging between 50 and 70%. The affected vessels included the middle cerebral arteries (MCAs), internal carotid arteries (ICAs), and the basilar artery (BA). Two interventional neuroradiologists (RA, SA) reviewed the clinical electronic medical records from MRI/MRA and MR vessel wall imaging to confirm ICAD-related stenoses.

We also included data from 10 healthy volunteers (6 females). Informed written consent was obtained from all participants in this study. A summary of the demographic and physiological data for patients and volunteers is shown in Table 1. Hereafter, we refer to this cohort as the ICAD-HV dataset.

**Table 1:** Median age, BMI, and average heart rate (maximum and minimum values) for the control and ICAD groups

Parameter	Controls (*n* = 10)	ICAD (*n* = 25)
Age (years)	27	64
	(19–35)	(34–85)
BMI (kg/m^2^)	30.30	27.99
	(19.37–38.73)	(21.91–41.29)
Heart Rate (bpm)	90.6	73.8
	(72.6–121.2)	(63.0–115.2)

#### MRI acquisitions

Patients and volunteers were scanned using a clinical MRI protocol designed for intracranial vascular assessment. This protocol included a gradient-echo 3D ToF-MRA sequence to visualize vascular anatomy, and an ECG-triggered intracranial 4D flow MRI sequence to capture blood flow dynamics. The 4D flow MRI sequence used a dual-velocity encoding acquisition (dual-VENC) and was accelerated using PEAK GRAPPA with an acceleration factor of *R* = 5, as described in  [32]. All scans were performed at 3T (Siemens MAGNETOM Skyra, Erlangen, Germany; and Siemens MAGNETOM Prisma Fit) using a 20-channel head/neck coil (Siemens, Erlangen, Germany). The detailed scan parameters for 3D ToF-MRA and 4D flow MRI are provided in Table 2.

**Table 2:** Scan parameters for 3D ToF-MRA and dual-VENC 4D flow MRI in the control and ICAD groups

3D ToF-MRA Parameters		
Parameters	Controls	ICAD
TR [ms]	22	21
TE [ms]	3.42	3.4
Voxel size [mm]	0.52	0.52
Slice thickness [mm]	0.50	0.50
Flip angle [^∘^]	17	17
Scan time [min]	4–5	4–5
Siemens MR system	Prisma Fit	Skyra

Dual-VENC 4D flow MRI Parameters		
Parameters	Controls	ICAD
TR [ms]	5.9	6.1–6.2
TE [ms]	3.25	3.4
Temporal resolution [ms]	82.6	42.7 - 86.8
Voxel size [mm]	0.982	0.978–1.146
Slice thickness [mm]	1.0	1.0–1.2
Number of slices	44	40–60
Number of cardiac phases	5–9	5–18
Flip angle [^∘^]	15	15
Low venc/high venc [m/s]	0.5–0.6 / 1.0–1.2	0.5–0.6 / 1.0–1.2
Siemens MR system	Prisma Fit	Skyra

### External validation

To assess the generalizability of the proposed labeling framework, we evaluated it on the publicly available ToF-MRA TubeTK dataset^1^ of healthy volunteers. Acquisition details are provided in  [33]. From the TubeTK dataset, we selected a subset of 20 cases (11 females; median age 34 years; range 20–48 years) with voxel size 0.51 × 0.51 × 0.8 mm^3^.

### Automated labeling

**Fig. 1:**
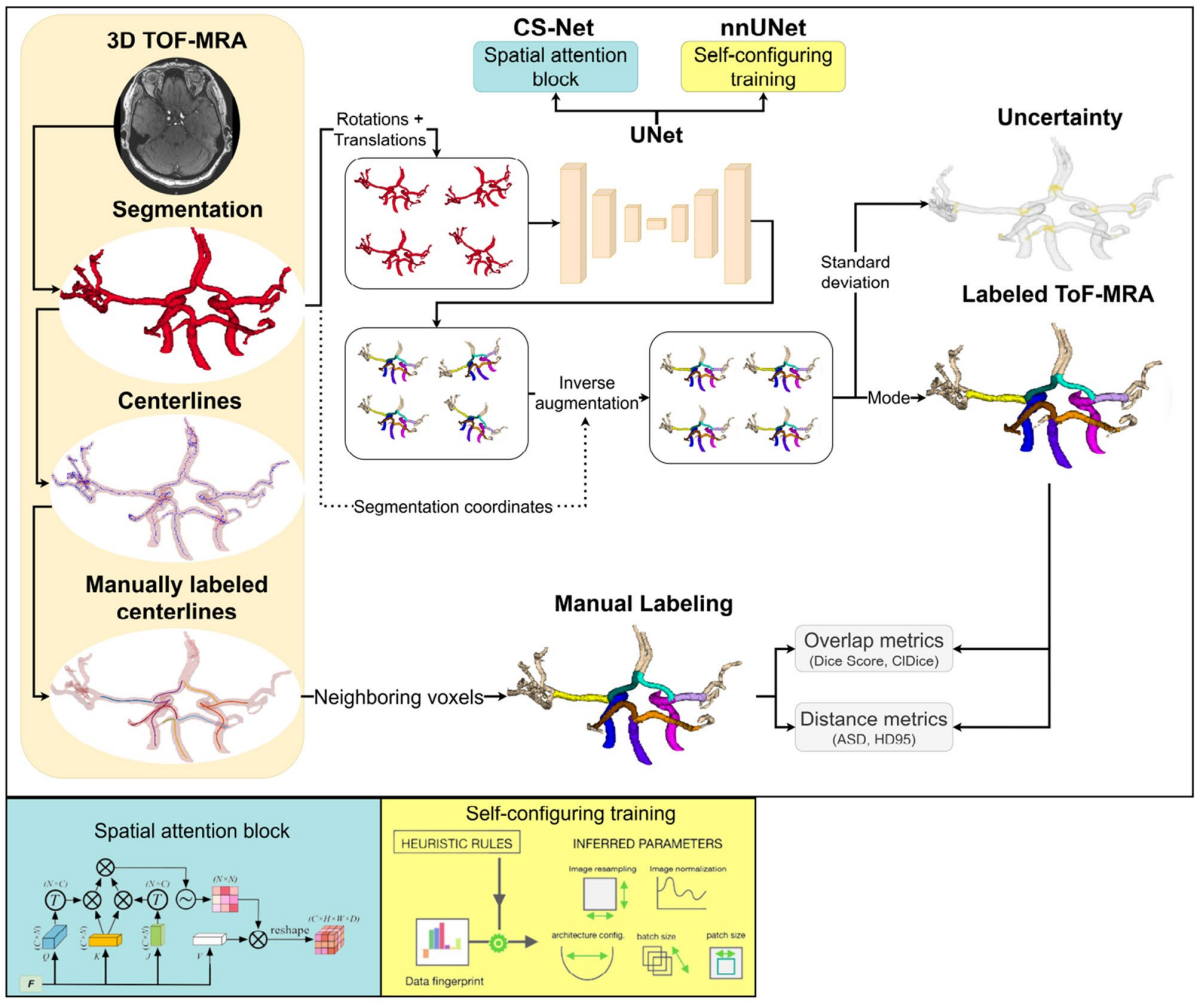
Overview of the automated labeling framework. 3D ToF-MRA images were used to segment intracranial arteries, followed by centerline extraction and manual labeling of nine arterial segments. Neighboring voxels of centerlines were labeled for voxelwise classification. Three UNet variants (UNet, CS-Net, and nnUnet) were trained to perform voxelwise classification directly from the segmentations. Test-time augmentation (TTA) was applied to estimate uncertainty. Labeling performance was evaluated using the Dice score, centerline Dice (clDice), average surface distance (ASD), and 95th percentile Hausdorff distance (HD95)

An overview of the automated labeling framework including preprocessing, labeling, and uncertainty quantification is shown in Fig. 1.

#### Data preparation

**Fig. 2:**
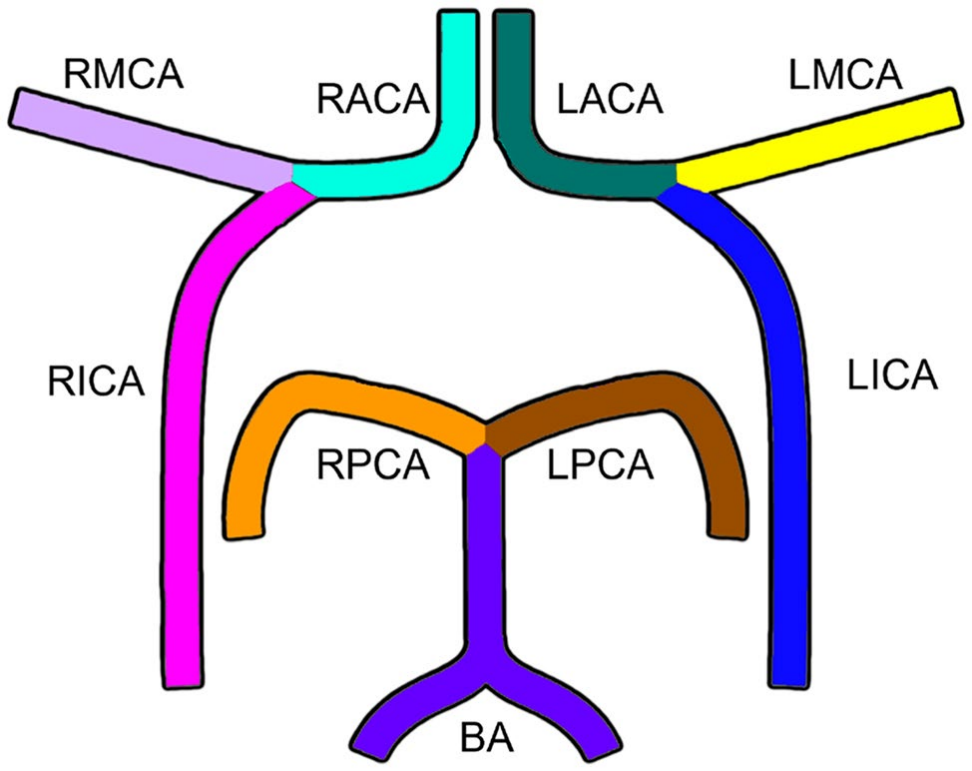
Schematic representation of the nine intracranial artery segments included in this study: basilar artery (BA); right and left internal carotid arteries (RICA, LICA); right and left middle cerebral arteries (RMCA, LMCA); right and left anterior cerebral arteries (RACA, LACA); and right and left posterior cerebral arteries (RPCA, LPCA)

We generated binary masks of the cerebral vasculature using semi-automatic thresholding applied to 3D ToF-MRA images. As in previous work  [8], an in-house algorithm was employed to automatically extract the centerlines of the cerebral vasculature. The identified centerlines were then manually labeled according to the anatomical section of the vessels. We focused our study on annotating nine major vessels of the Circle of Willis: the basilar artery (BA), right and left internal carotid arteries (RICA and LICA), right and left middle cerebral arteries (RMCA and LMCA), right and left anterior cerebral arteries (RACA and LACA), and right and left posterior cerebral arteries (RPCA and LPCA). These vessels are illustrated in Fig. 2. Although ToF-MRA can depict more arteries than those selected for annotation, our focus was motivated by the intended integration with 4D Flow MRI. This technique has been primarily validated in arteriovascular sections proximal to, and including, the Circle of Willis  [12, 34]. Smaller or more peripheral vessel segments become increasingly sensitive to trade-offs between spatial resolution, acquisition time, image noise, and partial-volume effects  [35].

To generate voxelwise ground truth labels, we used a 7 × 7 × 7 voxel neighborhood centered on each centerline position. Each voxel within this neighborhood was assigned the label of its closest centerline point. This procedure produced multi-class masks for each binary ToF-MRA segmentation. Voxels without a centerline point within their 7 × 7 × 7 neighborhood were classified as “non-annotated”. This neighborhood size was empirically selected to cover the diameter of all vessels, ensuring that boundary voxels were not left unannotated. Additionally, near the terminal centerline points of each vessel, the neighborhood was reduced to 5 × 5 × 5 to avoid assigning annotated voxels to regions that should remain unannotated.

#### Network architectures

Our work evaluated three state-of-the-art UNet variants for semantic segmentation, each chosen with a distinct objective: 1) automate preprocessing, hyperparameter tuning, and training strategies; 2) integrate advanced architectural refinements; and 3) benchmark against previous work.

1. **Self-configuring UNet**  [29] (nnUNet): nnUNet is a framework that automatically configures the full segmentation pipeline for a given dataset. It derives a dataset “fingerprint” from the training data (e.g., intensity distribution, voxel spacing, image dimensions, and label characteristics) and applies a set of rule-based decisions to select key preprocessing steps (e.g., resampling) and training settings (e.g., image resampling strategy, network topology). In prior large-scale evaluations, nnUNet outperformed many specialized task-specific methods, and it was the winning approach in the Medical Segmentation Decathlon  [36]. These results motivate its use as a robust, reproducible, and high-performing algorithm.
2. **Channel and Spatial Attention Network**  [13] (CS-Net): CS-Net enhances the standard UNet architecture by integrating channel and spatial attention mechanisms. These mechanisms enable the network to better capture fine-grained details and contextual information, making this variant particularly well-suited for tasks requiring precise segmentation of curvilinear structures. CS-Net outperformed 3D baselines, such as UNet, achieving higher vessel detection rates with fewer false positives. Initially designed for segmentation, we changed the number of classes in the last layer to adapt it to our labeling task.
3. **UNet**  [37] (baseline): This variant replaces the standard UNet encoder with a residual encoder, incorporating skip connections at each encoder layer to improve gradient flow and mitigate the vanishing gradient problem  [38]. As it represents the architecture used in recently published work on automated intracranial labeling  [20, 22], this UNet variant serves as our baseline model.

#### Preprocessing

Two preprocessing pipelines were implemented to ensure that the input images align with the input formats required by the UNet, CS-Net, and nnUNet setups.

1. **Scaling with zero-padding and cropping (UNet and CS-Net)**: Convolutional networks typically require input images of a fixed size. To handle images of varying dimensions, we first extracted a bounding box that encapsulates each segmentation, adding an empirically chosen 15% zero-padding margin along each dimension. Each image was also scaled to match the largest dimension of the target shape, while preserving the original aspect ratio. This ensured that the anatomical structures were not distorted. Subsequently, we applied cropping to adjust the image to the exact target dimension. For this application, we used a target dimension of $128\times256\times256$ pixels.
2. **Patch inference (nnUNet)**: As part of the nnUNet preprocessing pipeline, the proposed patch-based approach was utilized directly, allowing for input images of different sizes. Patches with dimensions equal to the median shape of the dataset ($80\times224\times160$ pixels) were extracted using a sliding window strategy with half-patch size overlap. Gaussian patch weighting was applied, with standard deviation equal to 0.125 of each dimension  [39]. This allowed the model to predict on images of varying dimensions without resizing.

#### Data augmentation

To avoid overfitting, we applied spatial transformations to the input segmentations during training. Specifically, we applied random rotations within ±18^∘^ around each axis, random translations within ±5 voxels along each axis, and random scaling with a factor sampled uniformly from 0.9 to 1.1.

#### Loss function

For *C* classes and *N* voxels, let $p_{i,c}$ be the predicted probability and $g_{i,c}\in\{0,1\}$ be the one-hot ground truth. The Dice coefficient per class is

1 $$\mathrm{Dice}(c)=\frac{2\sum_{i}^N p_{i,c} g_{i,c}}{\sum_{i}^N p_{i,c}+\sum_{i}^N g_{i,c}},$$

and the Dice loss is the complement averaged over classes:

2 $$L_{\mathrm{Dice}}=1-\frac{1}{C}\sum_{c=1}^{C}\mathrm{Dice} (c).$$

The cross-entropy loss is defined as :

3 $$L_{\mathrm{CE}}=-\frac{1}{N}\sum_{i=1}^{N}\sum_{c=1}^{C} g_{i,c}\log(p_{i,c}).$$

We utilized a hybrid loss that includes cross-entropy (*L*_CE_) and Dice (*L*_*Dice*_) losses with dynamic weights  [20], defined as

4 $$L=\left\{\begin{array}{cc}L_{\mathrm{CE}} & \text {epoch } \leq \beta \\\alpha L_{\mathrm{CE}}+(1-\alpha) L_{\text {Dice }} & \beta < \text {epoch } \leq \gamma \\0.9 L_{\text {Dice }} + 0.1L_{\mathrm{CE}}, & \gamma < \text {epoch } \leq \text {total }\end{array}\right.$$

where

5 $$\alpha=0.8\left(1-\frac{\mathrm{epoch}-\beta}{\gamma-\beta}\right)+0.1.$$

This loss and the accompanying weights ensure that during the initial training epochs ($\leq\beta$), the networks focus on minimizing the cross-entropy loss, leading to more stable convergence. Between epochs *β* and *γ*, the contribution of the two losses is balanced by the weight factor *α*, which gradually decreases as training progresses, shifting the emphasis from cross-entropy loss to Dice loss. Finally, after epoch *γ*, the loss stabilizes with a fixed weighting of 0.9 for Dice and 0.1 for cross-entropy, so the networks prioritize Dice loss, which, although less stable, can lead to more accurate predictions  [20].

#### Uncertainty quantification with modified test-time augmentation

We estimated the uncertainty using test-time augmentation (TTA)  [30]. Among uncertainty quantification methods for medical image segmentation, Monte Carlo (MC) dropout and deep ensembles are widely adopted  [28]. However, MC dropout performance is sensitive to implementation factors such as dropout rate, sampling scheme, and layer placement, which vary between architectures and influence uncertainty estimates  [40]. Deep ensembles provide robust uncertainty estimation by training multiple independent models, but at the cost of substantially increased computational demands. Because our study compares several architectures, we opted for TTA as a model-agnostic and training-free alternative.

TTA generates multiple slightly different inputs, each representing a plausible variation of the original data. Large variations in labeling across augmented versions indicate regions where the model is less confident, often due to intrinsic data variability from anatomical abnormalities, imaging artifacts, or annotation ambiguities. In our implementation, during inference, we applied random rotations (within ±18^∘^ per axis) and translations (within ±5 voxels per axis) to simulate realistic positional variations. For each acquisition, we generated seven predictions, inverted the spatial transformations, and computed the voxelwise standard deviation across the predictions as a measure of uncertainty. We also obtained final labels via voxelwise majority voting (mode) over the aligned augmented predictions.

Although rotations and translations are inherently invertible, interpolations are not and can introduce errors, particularly at label boundaries, which may distort uncertainty estimates in TTA. To mitigate this, we developed a coordinate-guided transformation method. For each point in the original image domain, we defined X, Y, and Z grids and extracted the coordinates corresponding to segmented voxels. Then, we applied the TTA transformations to these coordinates and rounded them. The round operation is equivalent to finding the closest coordinate to each voxel index in the transformed image domain. Each label is then paired with a coordinate, minimizing misassignments to the background. This approach significantly reduced interpolation errors, with only minor errors remaining at the label interfaces.

#### Ablation study of uncertainty-aware component

We performed an ablation study comparing inference without uncertainty-aware processing (single forward pass; No TTA) against our uncertainty-aware inference using the mode of test-time augmentations (Standard TTA and our Coordinate-guided TTA).

#### Validation of coordinate-guided label inversion

To evaluate the accuracy of the proposed coordinate-guided inversion used in test-time augmentation, we applied 100 random spatial transformations (rotations ±18^∘^ per axis, translations ±5 voxels per axis) to manually labeled data from one acquisition of a healthy volunteer. The inverted labels were reconstructed using either the standard affine inversion (nearest-neighbor interpolation) or the coordinate-guided method. We quantified the fraction of misassigned voxels after each inversion to assess accuracy.

### Experimental setup

To estimate the accuracy and stability of the three implemented networks, we performed a 5-fold stratified cross-validation. For each cross-validation iteration, 28 scans were used for training (80% of the data), and the remaining 7 for testing (20% of the data).

#### Network implementation

UNet and CS-Net were implemented using the MONAI open-source framework  [41], while nnUNet was implemented using its public library^2^. All implementations were built on the PyTorch deep learning framework  [42]. Training, testing, and uncertainty quantification scripts, as well as model weights, are publicly available^3^. Note that reproducing the training for nnUNet requires following the guidelines provided in  [39].

The UNet and CS-Net networks were trained using the Adam optimizer  [43], while nnUNet was trained with stochastic gradient descent (SGD). A linear learning rate scheduler was used for all optimizers, with parameters set specifically for each optimizer. For Adam, the initial learning rate was set to 0.001 and the final learning rate to 0.0001, while for SGD, the initial learning rate was 0.01 and the final learning rate was 0.001.

UNet and CS-Net were trained for 2000 epochs with *β* = 800 and *γ* = 1200, while nnUNet was trained for 1000 epochs with *β* = 400 and *γ* = 600 (Eq. 4). The number of training epochs was chosen to ensure stable convergence, defined as an increase in the Dice score of less than 10^−4^ over 10 consecutive epochs, as observed in the training curves. All networks were trained on an NVIDIA A100–SXM4-40GB GPU. The total training times were 23:30 (HH:MM) for UNet, 27:30 for CS-Net, and 38:05 for nnUNet.

Specific architectural details, including convolutional layers, activation functions, and additional refinements, can be found in Table 3.

**Table 3:** Architecture details of UNet, CS-Net, and nnUnet. The stride and kernel sizes have the same value for all dimensions

Parameter	UNet	CS-Net	nnUNet
Input size (pixels)	128x256x256	128x256x256	80x224x160
Layers	6	5	6
Channels size	(16, 32, 64, 128, 256, 512)	(16, 32, 64, 128, 256)	(32, 64, 128, 256, 320, 320)
Stride	2	2	2
Kernel size	3	3	3
Activation	PReLU	ReLU	LeakyReLU
Sliding window	No	No	Yes
Residual encoder	Yes	Yes	Yes
Spatial attention	No	Yes	No

#### Automated labeling evaluation

For evaluation, we considered the models at the last epoch of each training session. This approach prevents any bias toward better performing models in the test set for each cross-validation iteration.

Four metrics were used to evaluate automated labeling performance: the Dice score (Dice; Eq. 1), the centerline Dice (clDice) (Eq. 7), the average surface distance (ASD) (Eq. 8), and the 95th-percentile Hausdorff distance (HD95) (Eq. 9).

To assess topology preservation, we report clDice, which combines topology precision and sensitivity based on the skeletons (centerlines) of the segmentations:

6 $${t_{{\rm{prec}}}} = {{|{\rm{Sk}}(Y) \cap X|} \over {|{\rm{Sk}}(Y)|}},\,{t_{{\rm{sens}}}} = {{|{\rm{Sk}}(X) \cap Y|} \over {|{\rm{Sk}}(X)|}},$$

7 $$\mathrm{clDice} = \frac{2\, t_{\mathrm{prec}}\, t_{\mathrm{sens}}}{t_{\mathrm{prec}} + t_{\mathrm{sens}}},$$

where $\mathrm{Sk}(\cdot)$ denotes a 3D skeletonization operator.

Surface agreement was quantified using ASD and HD95. ASD is defined as:

8 $$\mathrm{ASD} = \frac{1}{|S_X| + |S_Y|} \left( \sum_{x \in S_X} d(x, S_Y) + \sum_{y \in S_Y} d(y, S_X) \right),$$

where *X* and *Y* denote the manually and automatically labeled regions, respectively; *S*_*X*_ and *S*_*Y*_ are their corresponding surfaces; and $d(a,S)$ is the minimum Euclidean distance from point *a* to surface *S*. Here, $|\cdot|$ denotes the number of sampled surface points.

Finally, HD95 quantifies near-worst-case boundary error while remaining robust to outliers:

9 $$\mathrm{HD95} = \mathrm{P}_{95}\Big(\{d(x,S_Y): x\in S_X\}\ \cup\ \{d(y,S_X): y\in S_Y\}\Big),$$

with $\mathrm{P}_{95}(\cdot)$ denoting the 95th percentile.

#### 4D flow MRI velocity analysis

To investigate whether anatomical labels could guide the analysis of corresponding 4D flow MRI datasets, we evaluated the agreement between velocity measurements derived from manual reference labels and those generated by the best-performing automated labeling network. Specifically, we assessed whether the average velocity at peak systole within anatomically labeled regions was consistent between manual and network-based labels.

To co-register ToF-MRA images with 4D flow MRI data, we used a semi-automatic MATLAB tool  [8] that performs rigid registration with built-in functions from the SPM12 toolbox (Statistical Parametric Mapping 12)  [44]. Following co-registration, both manually and automatically labeled ToF regions were downsampled by a factor of two to match the resolution of the 4D flow MRI data.

Vessel-specific average velocities at peak systole, obtained from manual and automated labeling, were then compared via Bland–Altman analysis and the Wilcoxon signed-rank test for statistical evaluation. A Benjamini-Hochberg correction was performed to adjust for multiple comparisons.

## Results

### Automated labeling

**Fig. 3:**
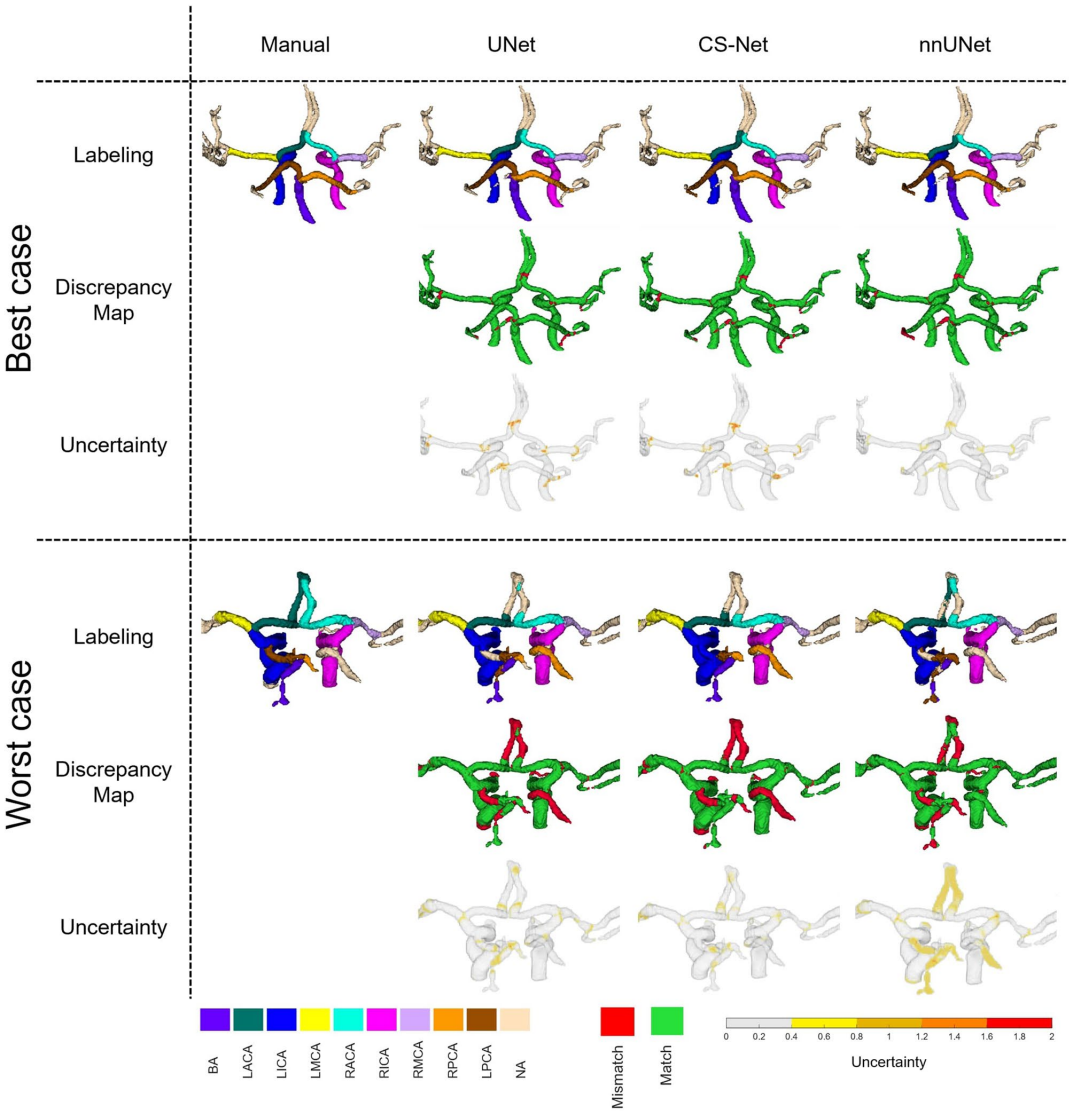
Automated labeling results for the best and worst-case patient data. For each case, the first row illustrates the manual labeling alongside the automated labeling predictions from UNet, CS-Net, and nnUnet. The second row highlights the match and mismatch regions between the manual and automated labeling. The third row visualizes uncertainty, measured by the standard deviation of test-time augmentation predictions

Figure 3 presents the automated labeling results for the best and worst cases in terms of the average Dice score across all networks.

In the best-case scenario all networks achieved accurate labeling of the vessels of interest with only minor errors occurring primarily at vessel bifurcations or connections with non-annotated segments. These mislabeled regions are highly correlated with areas of higher uncertainty.

In contrast, for the worst-case scenario, three regions showed some problems:

- **RPCA misclassification**: UNet and CS-Net incorrectly labeled the RPCA segment, probably due to its anatomical similarity and proximity to a variant vessel branching from the RICA (a common hyperplastic posterior communicating artery variant  [45]). While UNet and CS-Net failed to capture this uncertainty, nnUNet correctly classified this region as non-annotated with a relatively high uncertainty, recognizing it as an anatomical variant.
- **BA stenosis**: All networks struggled to label the BA segment in a patient with severe stenosis. UNet and CS-Net partially captured the uncertainty associated with this error, while nnUNet showed high uncertainty throughout the stenotic BA.
- **ACA variability**: All networks misclassified upper ACA segments due to inconsistencies in manual labeling protocols, particularly to delineate terminal portions of smaller vessels. These variations were consistently reflected in the uncertainty estimates across all networks.

Table 4 shows the quantitative evaluation of the labeling performance. Consistently strong performance is observed across all networks, with nnUNet achieving the highest overall accuracy. For Dice, nnUNet demonstrated superior performance (0.93) compared with CS-Net (0.91) and UNet (0.90), with notable improvements in the LMCA (0.94 vs. 0.93/0.92) and RACA (0.88 vs. 0.82/0.82) segments. Although all networks excelled at labeling larger vessels, such as LICA and RICA (Dice > 0.98), smaller vessels, including the ACA and PCA segments, proved more challenging, showing lower agreement between methods.

**Table 4:** Cross-validation metrics for the ICAD-HV dataset (*n* = 25 ICAD patients and *n* = 10 healthy volunteers). Dice, clDice, HD95, and ASD for UNet, CS-Net, and nnUnet, reported for each vessel. For each vessel and metric, the best method is bolded (highest for Dice/clDice; lowest for HD95/ASD)

Metric	Network	Vessel									
		BA	LACA	LICA	LMCA	LPCA	RACA	RICA	RMCA	RPCA	Average
Dice	UNet	0.93	0.85	**0.99**	0.92	0.88	0.82	**0.98**	0.86	0.90	0.90
	CS-Net	**0.94**	0.86	**0.99**	0.93	0.89	0.82	**0.98**	**0.88**	0.89	0.91
	nnUNet	**0.94**	**0.87**	**0.99**	**0.94**	**0.91**	**0.88**	**0.98**	**0.88**	**0.92**	**0.93**
clDice	UNet	0.95	0.88	0.97	0.96	0.88	0.85	0.97	0.89	0.90	0.91
	CS-Net	**0.96**	0.88	**0.99**	**0.97**	0.90	0.85	0.97	0.91	0.88	0.92
	nnUNet	0.95	**0.89**	0.98	**0.97**	**0.93**	**0.89**	**0.98**	**0.92**	**0.92**	**0.94**
HD95 (mm)	UNet	3.40	9.56	2.01	4.92	11.51	8.83	1.98	8.54	8.91	6.61
	CS-Net	3.75	9.58	**0.39**	**3.69**	10.10	8.35	**1.62**	**5.30**	11.19	5.96
	nnUNet	**2.74**	**8.18**	0.70	**3.69**	**7.13**	**7.20**	1.65	5.34	**4.73**	**4.51**
ASD (mm)	UNet	0.31	0.62	0.16	0.37	0.73	0.50	0.16	0.66	**0.65**	0.46
	CS-Net	0.28	**0.53**	0.05	0.20	0.69	**0.46**	0.12	0.36	0.73	0.38
	nnUNet	**0.23**	0.70	**0.04**	**0.17**	**0.43**	0.49	**0.11**	**0.33**	0.72	**0.35**

Regarding topology preservation, as quantified by clDice, nnUNet achieved the highest average clDice score (0.94) outperforming CS-Net (0.92) and UNet (0.91). For large vessels, such as LICA and BA, CS-Net showed superior clDice performance, whereas for smaller vessels, including RPCA, LPCA, and RACA, nnUNet exhibited better topology preservation.

In terms of surface metrics, nnUNet yields the lowest average HD95 (4.51 mm) and ASD (0.35 mm), outperforming CS-Net (HD95: 5.96 mm; ASD: 0.38 mm) and UNet (HD95: 6.61 mm; ASD: 0.46 mm), indicating both fewer outlier boundary errors (HD95) and better mean boundary alignment (ASD). The largest differences are seen in the distal PCA segments (e.g., LPCA HD95: 7.13 mm vs. 10.10/11.51; ASD: 0.43 mm vs. 0.69/0.73) and RMCA (ASD: 0.33 mm vs. 0.36/0.66). Nevertheless, there are vessel-specific exceptions: CS-Net achieves lower HD95 for RICA (1.62 mm vs. 1.65) and RMCA (5.30 mm vs. 5.34), and the lowest ASD for LACA (0.53) and RACA (0.46).

For a single patient case in one artery (RPCA), the ground truth was so small that nnUNet did not produce a prediction, resulting in a Dice value of 0 and $\infty$ ASD. To quantify ASD statistics, we replaced the ASD value of this specific case with the maximum ASD observed among all network predictions.

**Fig. 4:**
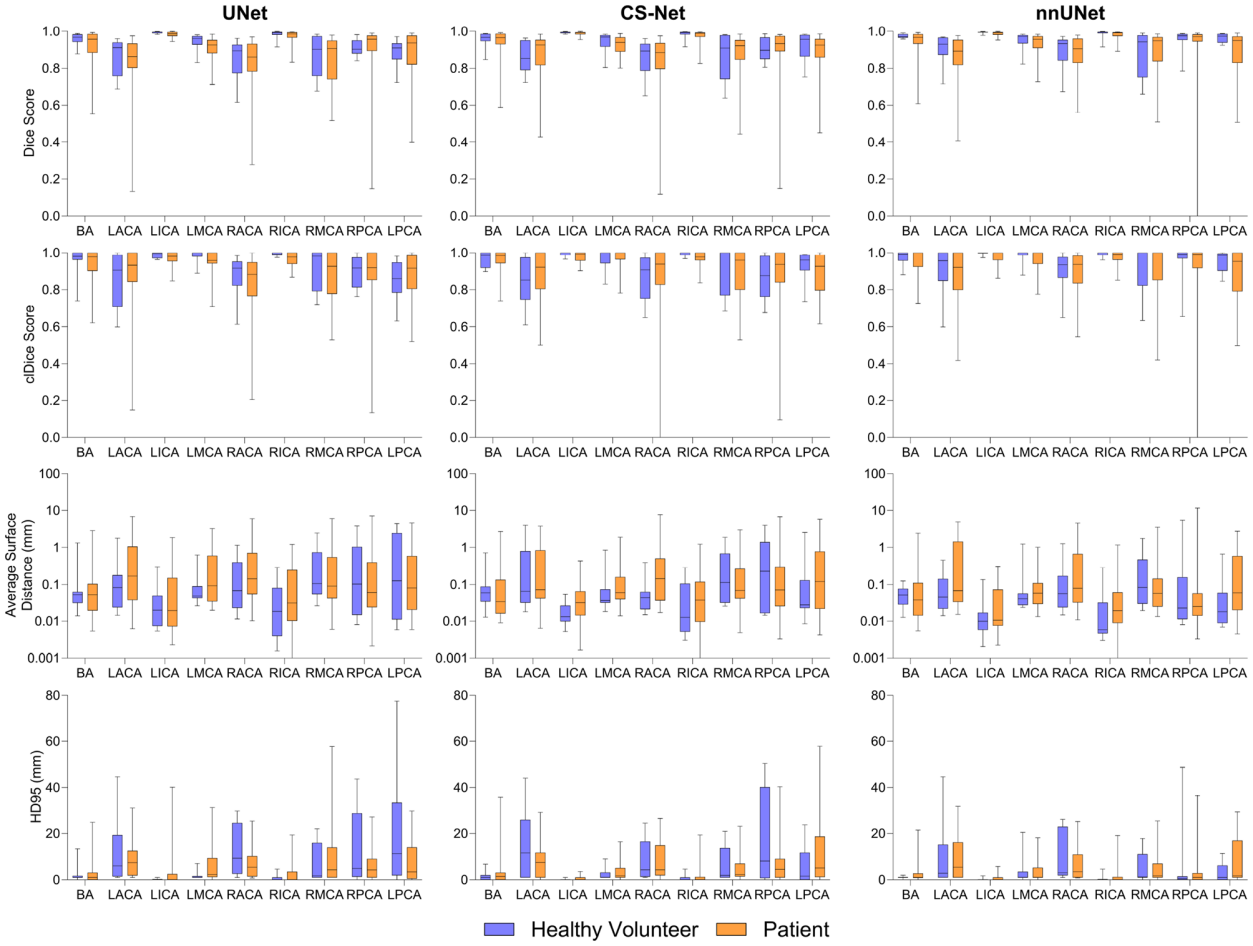
Boxplot analysis of Dice score, centerline Dice, average surface distance, and 95th percentile of Hausdorff distance (HD95) distributions across networks comparing labeling performance between healthy volunteers (*n* = 10) and patients (*n* = 25) (excluding external validation on TubeTK dataset). Whiskers represent the full data range (minimum to maximum values)

Boxplots (Fig. 4) illustrate differences between healthy volunteers and patients, as well as between networks. For all networks, the median Dice scores did not show significant differences between the healthy and patient groups, although the variability of the Dice score tended to be higher in patients, particularly in smaller or more complex vessels such as RPCA and LPCA. clDice showed a similar trend, medians remain high for large vessels (e.g., ICA/BA), and variability was higher in smaller branches for patients.

In contrast, the surface-distance metrics (ASD and HD95) metrics revealed differences between volunteers and patients, although the pattern of these differences varied across networks. For UNet, ASD values were lower in patients for 5 of the 9 vessels (BA, LICA, RACA, RMCA, RPCA, and LPCA). For CS-Net, ASD was lower in patients for 3 of the 9 vessels (BA, RMCA, and RPCA). For nnUNet, ASD was lower in patients for 2 of the 9 vessels (BA and RMCA). For HD95, the patient group showed lower values for fewer vessels: for UNet, HD95 was lower in patients for 3 of the 9 vessels (RACA, RPCA, and LPCA). For CS-Net, HD95 was lower in patients for 2 of the 9 vessels (LACA and RPCA). For nnUNet, HD95 was higher in patients for all vessels.

Table 5 shows the ablation study on uncertainty, comparing inference with and without TTA. Overall, introducing the uncertainty-aware inference yields small but consistent improvements in labeling metrics for the best performing models (CS-Net and nnUNet). Comparing Coordinate-guided TTA with Standard TTA shows no significant differences in the mean performance metrics.

**Table 5:** Average Dice, clDice, HD95, and ASD for CS-Net and nnUnet across test-time augmentation (TTA) strategies. The best value across TTA strategies is bolded (highest for Dice/clDice; lowest for HD95/ASD)

Network	No TTA				Standard TTA				Coordinate-guided TTA			
	Dice	clDice	HD95	ASD	Dice	clDice	HD95	ASD	Dice	clDice	HD95	ASD
CS-Net	**0.91**	**0.92**	7.28	0.52	**0.91**	**0.92**	**5.91**	**0.37**	**0.91**	**0.92**	5.96	0.38
nnUNet	0.92	0.93	4.73	0.39	0.92	**0.94**	**4.48**	**0.35**	**0.93**	**0.94**	4.51	**0.35**

External validation on the TubeTK dataset (*n* = 20 healthy volunteers; Table 6) demonstrated generalizability beyond the ICAD-HV cohort. CS-Net and nnUNet achieved the highest overall overlap (average Dice: 0.87 for both) compared to UNet (0.84). nnUNet provided the greatest topology preservation and the lowest near worst boundary error (average clDice: 0.87; HD95: 5.85 mm), while CS-Net achieved the lowest mean surface error (ASD:0.39 mm, versus 0.42 mm for nnUNet and 0.86 mm for UNet). Overall, labeling performance was higher in larger vessels (e.g., BA and ICA with Dice ≥ 0.89) and decreased in smaller distal branches, particularly PCA segments.

**Table 6:** External validation metrics for the TubeTK dataset (*n* = 20 healthy volunteers). Dice, clDice, HD95, and ASD for UNet, CS-Net, and nnUnet, reported for each vessel. For each vessel and metric, the best method is bolded (highest for Dice/clDice; lowest for HD95/ASD)

Metric	Network	Vessel									
		BA	LACA	LICA	LMCA	LPCA	RACA	RICA	RMCA	RPCA	Average
Dice	UNet	0.86	0.86	0.92	**0.80**	0.75	0.85	0.95	0.80	0.71	0.84
	CS-Net	**0.90**	0.85	**0.95**	**0.80**	0.78	0.86	**0.97**	**0.88**	0.81	**0.87**
	nnUNet	0.89	**0.91**	0.94	0.77	**0.81**	**0.89**	0.96	0.85	**0.82**	**0.87**
clDice	UNet	0.83	0.85	0.87	**0.79**	0.65	0.87	0.89	0.81	0.66	0.80
	CS-Net	**0.93**	0.81	**0.93**	0.78	**0.75**	0.88	0.94	**0.91**	**0.80**	0.86
	nnUNet	0.90	**0.94**	**0.93**	0.77	**0.75**	**0.94**	**0.95**	0.87	0.77	**0.87**
HD95 (mm)	UNet	12.07	3.68	11.11	8.91	12.14	5.64	8.24	7.98	16.27	9.54
	CS-Net	**3.00**	9.12	**2.66**	8.15	13.02	6.80	1.51	**4.23**	**12.80**	6.73
	nnUNet	4.03	**1.99**	3.78	**8.10**	**12.06**	**2.94**	**1.46**	5.96	12.82	**5.85**
ASD (mm)	UNet	1.16	0.34	0.99	0.96	1.34	0.38	0.69	0.92	1.01	0.86
	CS-Net	**0.25**	0.91	0.23	0.26	**0.59**	0.47	0.11	**0.20**	**0.51**	**0.39**
	nnUNet	0.39	**0.14**	**0.22**	**0.25**	1.26	**0.08**	**0.10**	0.48	0.87	0.42

### Validation of coordinate-guided label inversion

**Fig. 5:**
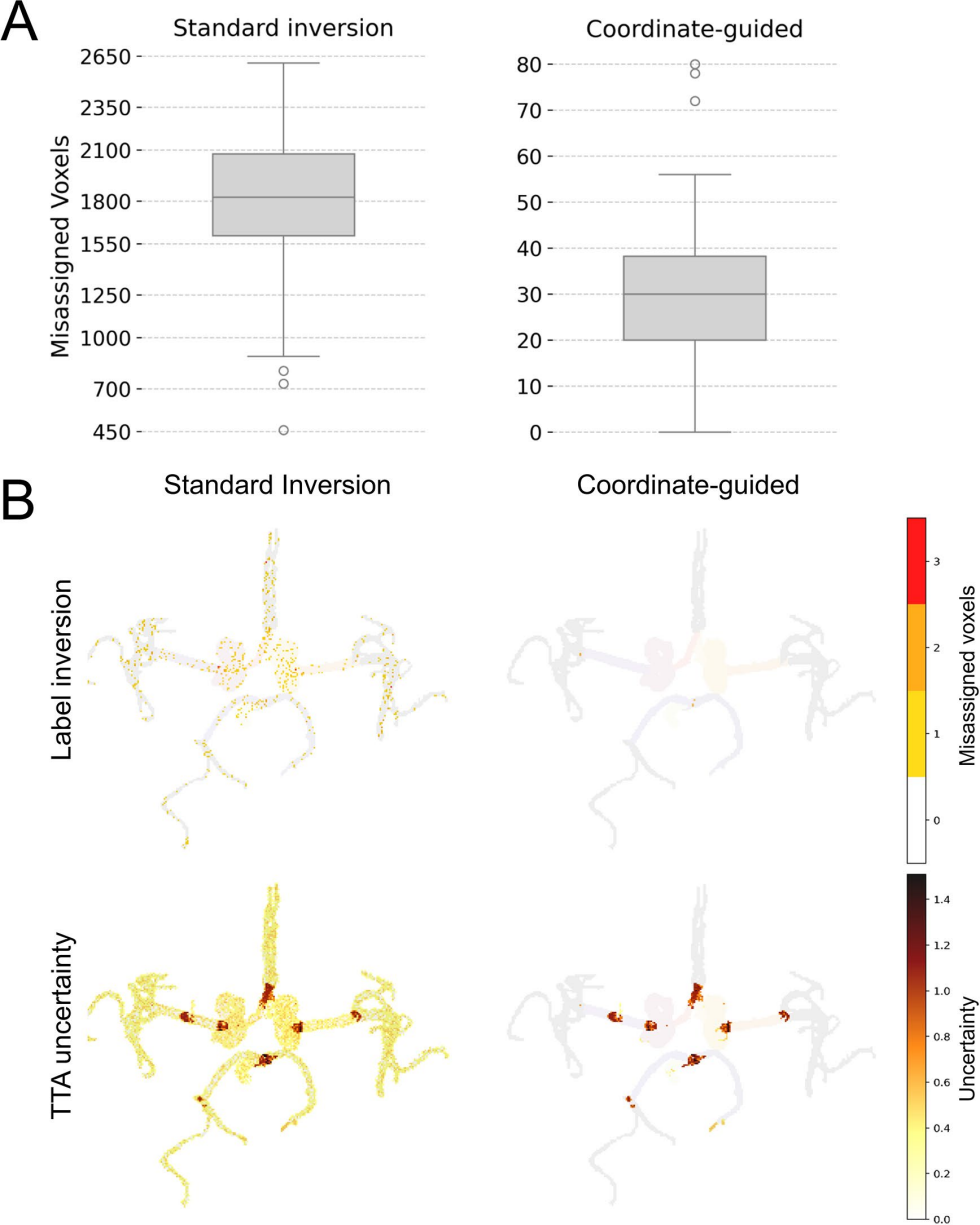
Validation of the coordinate-guided inversion and its impact on uncertainty estimation. (A) Distribution of label inversion errors across 100 random transformations applied to a manually labeled acquisition from a healthy volunteer. The left boxplot shows standard affine inversion (median $5.18\,\%$), while the right shows the coordinate-guided method (median $0.09\,\%$). (B) Comparison of both inversion methods for one representative random transformation. The first row illustrates label inversion results, where the sum of misassigned voxels along the slice direction is overlaid on the maximum intensity projection (MIP) of the labeled image. The second row shows the corresponding uncertainty maps, also displayed as MIPs along the slice direction

The coordinate-guided inversion substantially reduced label misassignments compared with the standard affine approach (0.09% ± 0.04% vs. 5.10% ± 1.17%, mean ± SD). Figure 5A shows that our method consistently maintained inversion errors below 0.2%, whereas the standard inversion reached up to 7%. Spatial error maps (Fig. 5B, first row) illustrate that interpolation artifacts from standard inversion were scattered along vessel borders, while the coordinate-guided method yields only minimal errors at label interfaces. The improved inversion also yielded more reliable uncertainty maps (Fig. 5B, second row), especially at segmentation boundaries where interpolation artifacts previously inflated uncertainty.

### 4D flow MRI analysis

**Fig. 6:**
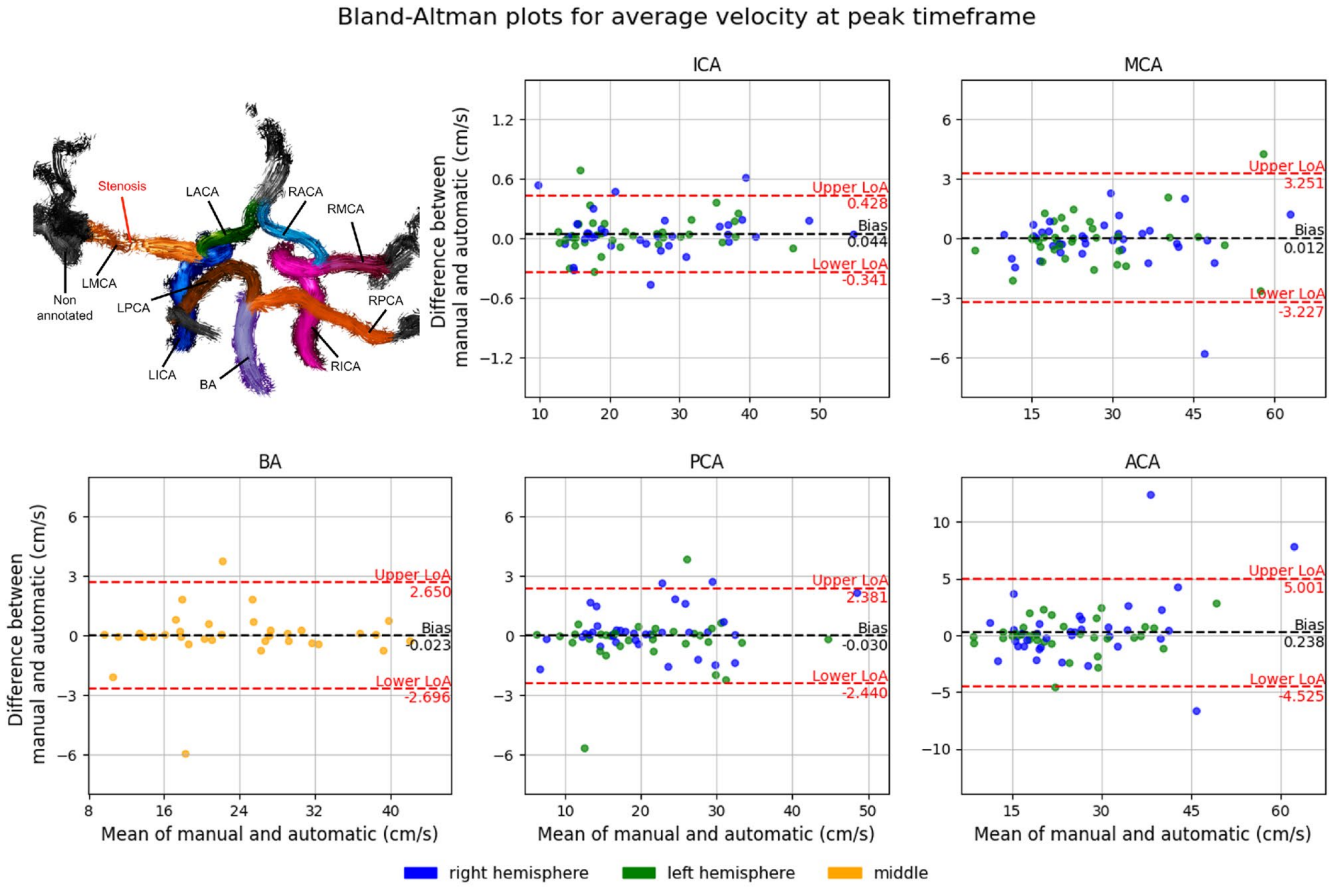
The upper left corner displays streamlines of different artery segments using automated labeling in a patient with severe stenosis in the left middle cerebral artery (LMCA). In the remaining figures, Bland-Altman plots compare the percentage differences in average velocity at peak systole between manual and automated labeling using nnUnet. Bias and limits of agreement are shown for each artery group: internal, middle, posterior, and anterior cerebral arteries (ICA, MCA, PCA, and ACA, respectively)

Figure 6 presents Bland-Altman plots for the 4D flow MRI analysis, comparing the average velocity at the peak systole timeframe between manual and automated labeling using the best-performing network (nnUNet). ICAs demonstrated the smallest differences between manual and automatically labeled vessels, with agreement limits ranging from −0.34 cm/s to 0.43 cm/s (−2.1% of the mean to 2.4% of the mean, respectively). The BA also exhibited relatively small differences, although a few outliers widened its limits of agreement from −2.70 cm/s to 2.65 cm/s (−12.4% of the mean to 12.8% of the mean). Larger discrepancies were also observed in the smaller arterial groups including MCAs (limits of agreement: −3.23 cm/s to 3.25 cm/s, equivalent to −10.7% to 9.4% of the mean), PCAs (limits of agreement: −2.44 cm/s to 2.38 cm/s; equivalent to −15.5% to 14.1% of the mean), and ACAs (limits of agreement: −4.53 cm/s to 5.00 cm/s; equivalent to −15.9% to 17.6% of the mean).

Table 7 presents Bland-Altman individual limits of agreement and average differences for all intracranial segments. Overall, the average differences were less than 2 cm/s for all segments, with 7 out of 9 segments showing an average absolute difference below 1 cm/s. When expressed as a percentage of the mean velocity, the average absolute differences ranged 0.54% (RICA) to 6.31% (LACA). Statistical analysis revealed no significant differences between manual and automatically labeled vessels for any of the derived average velocities.

**Table 7:** Width of limits of agreement (LoA), average absolute differences, and *p*-values for average velocity differences between manual and automated labeling, calculated per vessel using the Wilcoxon signed-rank test. *p*-values are shown before and after Benjamini-Hochberg correction to control for multiple vessel comparisons

Vessel	Abs. difference (cm/s)	Abs. difference (% of mean)	Width of LoA (cm/s)	Width of LoA (% of mean)	p-value	*p*-value (corrected)
BA	0.585	2.490	4.863	20.704	0.259	0.776
LACA	1.693	6.306	12.065	44.931	0.942	0.968
LICA	0.155	0.605	0.805	3.142	0.057	0.501
LMCA	0.799	2.833	5.712	20.259	0.704	0.968
RACA	1.005	4.213	5.789	24.267	0.919	0.968
RICA	0.125	0.540	0.752	3.243	0.369	0.806
RMCA	1.000	3.711	6.209	23.043	0.968	0.968
RPCA	0.621	3.028	7.096	34.598	0.447	0.806
LPCA	0.591	2.828	4.041	19.342	0.158	0.713

## Discussion

This study investigated the application of deep learning, specifically UNet-based architectures, for automated anatomical labeling of the major intracranial arteries from 3D ToF-MRA data. Unlike previous works that focused solely on labeling accuracy, our framework integrates uncertainty quantification to improve interpretability and reliability. We estimated the uncertainty using a coordinate-guided test-time augmentation (TTA) strategy, which effectively highlighted regions prone to labeling errors. The UNet baseline used in this study was a variant incorporating a residual encoder and a hybrid CE+Dice objective function with dynamically scheduled weights. These adaptations, established in prior work, have been shown to improve performance for ToF-MRA vascular labeling  [20]. Our findings demonstrate that, while architectural refinements such as channel and spatial attention blocks in CS-Net offered improved performance compared to the baseline UNet, the self-configuring nnUNet framework yielded the most accurate and robust labeling results. Validation through 4D flow MRI velocity analysis confirmed that automated labels generated by the best performing network (nnUNet) provide a reliable basis for subsequent hemodynamic assessment.

External validation on TubeTK demonstrates that nnUNet and CS-Net maintain robust generalization performance, supporting the applicability of the proposed uncertainty-aware labeling framework beyond the ICAD-HV distribution. Performance degradation is more pronounced in smaller vessels (e.g., ACA/PCA segments) than in larger vessels. The performance gap relative to ICAD-HV cross-validation is likely attributable to TubeTK’s lower and anisotropic resolution (0.51 × 0.51 × 0.8 mm^3^ vs. near-isotropic 0.52 × 0.52 × 0.5 mm^3^), which affects vessel conspicuity and amplifies partial-volume effects. Although random scaling during data augmentation improves robustness to geometric variability and partially mitigates resolution mismatch, resampling and interpolation cannot fully replicate partial-volume artifacts.

During the preparation of the manuscript of this work, Colombo et al. presented a nnUNet-based method for simultaneous segmentation of aneurysms and arteries from 3D ToF-MRA  [23]. Their results corroborate the suitability of nnUNet for cerebrovascular tasks, but their study did not compare alternative architectures or incorporate uncertainty analysis. By comparing UNet-based models and introducing a coordinate-guided uncertainty quantification, our study extends the nnUNet paradigm toward interpretable cerebrovascular analysis.

### Uncertainty quantification and clinical utility

Beyond accuracy, the integration of uncertainty quantification is crucial for the clinical translation of deep learning models. Because our model operates on vascular segmentations, the resulting uncertainty maps primarily reflect ambiguity in vessel morphology/topology and labeling consistency, rather than intensity variability or non-vascular context. Our implementation of TTA successfully captured regions where the model predictions were less confident. High uncertainty arises when an input image differs substantially from the types of image to which the model was exposed during training, for example, due to anatomical variations or disease-related changes. In these cases, applying geometric transformations, such as rotations or translations during TTA, results in unstable predictions. This occurs because the models are trained to be invariant to such transformations only within the training data distribution. When the input deviates from that distribution, their predictions become less stable.

As illustrated in Fig. 3, regions with higher uncertainty often corresponded to anatomical variations (e.g. hyperplastic posterior communicating artery variant affecting RPCA labeling), vascular alterations (e.g., BA stenosis), or inconsistencies arising from the manual labeling protocol itself (e.g., defining distal ACA segments). This provides clinicians with valuable information on the trustworthiness of automated labels in specific regions.

Moreover, our proposed coordinate-guided TTA strategy addresses interpolation errors during the inverse transformation step. By mapping transformed coordinates back to the original grid space, we minimized these artifacts, reducing label misassignments from near 5% to <0.1% compared to standard affine inversion and obtained cleaner uncertainty maps at the vessel boundaries.

TTA inference also showed small improvements in labeling metrics for CS-Net and nnUNet (Table 5), but no significant differences were observed between coordinate-guided TTA and standard TTA modes. However, our uncertainty validation (Fig. 5) shows a decrease in the number of misaligned voxels, particularly at vessel boundaries.

The uncertainty maps generated by UNet and CS-Net were less effective at delineating uncertain regions compared with those from nnUNet. Both networks tended to be overconfident when predicting structures with clear discrepancies from manual labels.

This observation aligns with the concept of confidence calibration, where the predicted probability should correspond to the true likelihood of correctness  [46]. For example, if a group of voxels are predicted as an artery with 0.85 probability, and 85% of such voxels are indeed correct, the model is considered well calibrated. Proper calibration is crucial for reliable TTA uncertainty estimation: an overconfident network produces near-zero uncertainty even in ambiguous areas, while an under-calibrated one overestimates uncertainty in regions where it should be confident.

To quantify calibration, we computed the expected calibration error (ECE)  [46], which measures the gap between predicted confidence and actual accuracy across binned predictions weighted by sample count. Using 15 bins, we obtained ECE values of $0.012 \pm 0.004$ for UNet, $0.008 \pm 0.004$ for CS-Net, and $0.006 \pm 0.003$ for nnUNet. These findings show that nnUNet had better calibration of its probabilities than UNet and CS-Net. This difference likely reflects nnUNet’s adaptive, data-driven configuration, which automatically optimizes network architecture for the dataset, whereas UNet and CS-Net were built using fixed, non-optimized settings. nnUNet’s more balanced feature distribution across layers likely contributed to its improved calibration and uncertainty performance.

### Labeling considerations

For nnUNet, we also highlighted performance differences between healthy volunteers and patients with ICAD (Fig. 4). In terms of ASD and HD95 CS-Net and nnUNet tended to perform worse on patients. This is likely due to the greater anatomical complexity and variability introduced by cerebrovascular diseases, such as stenoses or vascular morphology alterations, which pose a greater challenge for automated methods. Nevertheless, this was not a clear pattern for all vessels, suggesting that vascular morphology even in healthy volunteers is also challenging.

The median age of the ICAD group (64 years) differs significantly from that of the healthy volunteer cohort (27 years). However, age alone is unlikely to explain the observed differences. In healthy adults, ToF-MRA studies report only mild age-related changes in intracranial vessel morphology, predominantly affecting distal vessels  [33]. In contrast, recent evidence indicates that morphological characteristics of intracranial arteries are associated with atherosclerosis risk factors  [47]. Therefore, the observed differences are more likely attributed to disease-related vascular alterations rather than to age effects alone.

In contrast to previous approaches that operate directly on image intensity data, our pipeline performs anatomical labeling using pre-segmented vascular structures. This design choice reflects a deliberate focus on the spatial and geometric features of the vasculature, which are more relevant for accurate anatomical identification. The intensity-based features in ToF-MRA are often susceptible to artifacts, such as flow-related signal loss, inhomogeneities, or nonvascular enhancements, which can mislead the network and reduce its generalizability across diverse datasets  [48]. By decoupling the labeling task from the raw image intensities and instead using binary segmentations as input, the network is encouraged to learn from the morphology and topology of the vessels themselves. Furthermore, excluding intensity features helps prevent the propagation of undesired biases from intensity features in the uncertainty estimates, resulting in more meaningful and spatially grounded uncertainty maps.

### Validation for downstream 4D flow MRI analysis

The successful application of automated labels for 4D flow MRI velocity analysis (Fig. 6, Table 7) demonstrates the practical utility of our approach. Bland-Altman analysis revealed good agreement between velocity measurements derived from manual labels and those from nnUNet’s automated labels, with no statistically significant differences found for any vessel segment using the Wilcoxon signed-rank test. Although the agreement was excellent for larger vessels such as the ICAs, slight discrepancies were observed for smaller arteries (ACAs, PCAs, MCAs). This may reflect the slightly lower labeling accuracy in these segments, potential partial-volume effects, or minor residual co-registration inaccuracies. Nonetheless, the overall small average differences (<2 cm/s absolute difference for all segments, <1 cm/s for 7 out of 9 segments) suggest that the labeling of nnUNet provides a robust foundation for automated hemodynamic quantification, reducing the need for time-consuming manual delineation.

### Limitations

Despite promising results, our study has some limitations. First, our method relies on an initial semi-automated segmentation step; any vessels missed or incorrectly segmented in this initial mask are not correctly labeled by the network. Errors in the initial segmentation will inevitably propagate to the final labeling. Second, the validation involving 4D flow MRI is subject to potential co-registration errors between the ToF-MRA (used for labeling) and the 4D flow MRI datasets, which could influence the velocity comparison. Third, the number of datasets (*n* = 35 for ICAD-HV and *n* = 20 for TubeTK) was relatively limited, covering healthy volunteers and ICAD patients from a single MRI vendor. Although 5-fold cross-validation and the external validation provide a measure of robustness, evaluation on larger, multi-center datasets with a wider range of pathologies is needed to fully assess generalizability. Nevertheless, for functional hemodynamic assessment, large-scale intracranial 4D flow MRI datasets remain rare; therefore, the cohort used in this study is appropriate for the 4D Flow MRI validation.

### Future directions

Several promising directions can be pursued to extend our work. Calibration error metrics showed that nnUNet’s better uncertainty estimates were related to lower calibration error, but these errors can also be formulated as differentiable loss functions to train networks to predict even more reliable uncertainty estimates  [49]. In terms of architecture, building on previous studies that investigated architectural modifications within the nnUNet framework  [50], future research could explore integrating the attention mechanisms used in CS-Net into the self-configuring nnUNet pipeline. This approach may further improve performance by combining nnUNet’s robust automated pipeline with architectural innovations. The resulting vessel labels could also serve as reliable inputs for downstream tasks, including automated centerline labeling, contributing to a fully automated pipeline for cerebrovascular hemodynamic analysis. Furthermore, applying transfer learning to adapt the labeling model directly to 4D flow phase-contrast MRA (PC-MRA) data could eliminate the need for co-registration and potentially improve the accuracy of velocity quantification within automatically labeled vascular regions.

## Data Availability

The data used in this work is not publicly available due to privacy constraints but can be requested from the corresponding author.

## Footnotes

1 TubeTK dataset.

2 https://pypi.org/project/nnunetv2/

3 https://github.com/JavierBZ/IC-UNet

## Code availability

Training, testing, and uncertainty quantification scripts, as well as model weights, are publicly available at https://github.com/JavierBZ/IC-UNet.
